# Plasma exosomal miR-320d, miR-4479, and miR-6763-5p as diagnostic biomarkers in epithelial ovarian cancer

**DOI:** 10.3389/fonc.2022.986343

**Published:** 2022-12-14

**Authors:** Shiwen Wang, Xingguo Song, Kangyu Wang, Baibing Zheng, Qinghai Lin, Miao Yu, Li Xie, Liang Chen, Xianrang Song

**Affiliations:** ^1^ Department of Clinical Laboratory, Shandong Cancer Hospital and Institute, Shandong First Medical University and Shandong Academy of Medical Sciences, Jinan, Shandong, China; ^2^ Department of Clinical Laboratory, Shandong Provincial Third Hospital, Shandong University, Jinan, Shandong, China; ^3^ Department of Gynecological Oncology, Shandong Cancer Hospital and Institute, Shandong First Medical University and Shandong Academy of Medical Sciences, Jinan, Shandong, China; ^4^ Shandong Provincial Key Laboratory of Radiation Oncology, Shandong Cancer Hospital and Institute, Shandong First Medical University and Shandong Academy of Medical Sciences, Jinan, Shandong, China

**Keywords:** exosomes, miRNAs, epithelial ovarian cancer, diagnosis, biomarkers

## Abstract

**Background:**

Exosomal miRNA had been proved as the promising biomarkers for multiple cancers including epithelial ovarian cancer (EOC). This study aimed to validate the diagnostic accuracy of exosomal miR-320d, miR-4479, and miR-6763-5p for EOC.

**Materials and methods:**

Exosomes isolated from the plasma by ultracentrifugation were verified using TEM, qNano and western blot. MiRNAs sequencing was used to screen out the differential exosomal miRNAs and miR-320d, miR-4479, and miR-6763-5p were selected as candidates, which were further verified by RT-qPCR in 168 healthy donors and 161 primary EOC patients. Besides, the diagnostic accuracy of these three exosomal miRNAs were evaluated using the receiver operating characteristic curve (ROC).

**Results:**

MiRNAs sequencing revealed 95 differential exosomal miRNAs between EOC patients and healthy donors. Subsequently, exosomal miR-320d, miR-4479, and miR-6763-5p were significantly down regulated in EOC patients compared with healthy controls and benign patients. More importantly, these three miRNAs could serve as circulating diagnostics biomarkers for EOC, possessing areas under the curve (AUC) of 0.6549, 0.7781, and 0.6834, respectively. Moreover, these three exosomal miRNAs levels were closely associated with lymph node metastasis, meanwhile exosomal miR-320d and miR-4479 expression was related to tumor stage.

**Conclusion:**

Exosomal miR-320d, miR-4479, and miR-6763-5p might serve as potential biomarkers for EOC.

## Introduction

Epithelial ovarian cancer (EOC) is the most common cause of death from gynecological cancers (1, 2). In 2020, 207,252 women died of ovarian cancers worldwide (3). Due to the lack of diagnostic methods with high sensitivity and specificity, most EOC patients are not detected until the advanced stage (2). Therefore, it is very essential to develop reliable and inexpensive biomarkers for the diagnosis and treatment of ovarian cancer, such as liquid biopsy by detecting platelets, circulating cells, miRNAs, extracellular vesicles, and free DNA (cf DNA) in body fluid (4).

Exosomes are cell-derived vesicles with a diameter of 30 to 150 nm (5), its components, including nucleic acids, proteins, and lipids, are heterogeneous (6), and different components reflect the type of cell that produced them and the origin of the cell (7). Meanwhile, exosomes can mediate intercellular communication by transferring functional mRNAs and miRNAs. Therefore, it exerts pleiotropic roles in tumorigenesis, immunosuppression, metastasis, angiogenesis, and drug resistance (8–10). Furthermore, mounting studies had shown that exosomes not only regulate the biological behavior of cancer but also play an important role in cancer diagnosis, especially in early diagnosis (11), thus emerging as promising biomarkers for cancer.

MiRNAs are endogenous short noncoding RNA (containing about 22 nucleotides) and part of the epigenome. It can regulate gene expression at the post-transcriptional level by binding to the 3′untranslated regions (3′-UTRs) of the target mRNAs (12, 13). Mounting papers had reported that miRNAs were involved in multiple biological procedures. For example, the miR-200 family and miR-205 regulated epithelial to mesenchymal transition by targeting ZEB1 and SIP1 in tumor metastasis (14). MiR-125b-5p inhibited the proliferation and metastasis of HCC through TXNRD1 and low miR-125b-5p was associated with poor prognosis in HCC patients (15). Under hypoxic conditions, miR-375 can inhibit autophagy by reducing the level of ATG7 and impairs viability of HCC cells (16). Moreover, miRNAs can be enriched in the exosomes and are not easy to be degraded by endogenous RNase (17). The aberrant expression of exosomal miRNA is observed in many cancer patients and is associated with tumorigenesis and progression (18). This provides the possibility for exosomal miRNAs as highly sensitive and non-invasive diagnostic biomarkers. Recently, some studies had reported the diagnostic role of exosomal miRNAs in cancers. For instance, the expression level of exosomal miR-2276-5p was significantly decreased in glioma patients as compared to non-glioma patients and glioma patients with lower expression of exosomal miR-2276-5p were correlated with poorer survival rates (19). MiR-5684 and miR-125b-5p were significantly downregulated in the serum exosomes of the NSCLC patients and can serve as promising diagnostic and prognostic biomarkers for NSCLC (20).

In our study, we screened differentially expressed exosomal miRNAs between EOC patients and healthy donors by miRNA sequencing. Consequently, miR-320d, miR-4479, and miR-6763-5p were selected and validated their expression differences in a large cohort. In addition, the relationship between the levels of three exosomal miRNAs and clinical data of EOC patients and diagnostic efficiency for EOC were analyzed. The results showed that exosomal miR-320d, miR-4479, and miR-6763-5p can serve as novel biomarkers for EOC.

## Methods and materials

### Patients and healthy donors

A total of 173 healthy donors, 166 EOC patients, and 34 benign disease patients were enrolled in this study between January 2021 and January 2022 at the Shandong Cancer Hospital and Institute. After plasma samples were collected, centrifuged at 2000 ×g for 10 minutes to remove haemocytes including red blood cells, white blood cells, and platelets, again at 12000 ×g at 4°C for 10 minutes to remove the cellular debris, and the supernatant was retained and stored at -80°C until ultracentrifugation. Tumor staging was performed according to the International Federation of Gynecology and Obstetrics (FIGO) staging system. The healthy donors did not present any tumors or other immune and metabolic diseases. All EOC and benign disease patients did not receive any anti-tumor treatment and suffered from any other endocrine, immune or metabolic diseases before peripheral blood collection. CA125 and HE4 of EOC patients were detected by Electrochemiluminescence (Roche e801, Basel, Switzerland). Detailed clinical data of EOC patients are summarized in **Table 1**.

**Table 1:** Characteristics of EOC patients for differentially expressed exosomal miR-320d, miR-4479, and miR-6763-5p.

Characteristic No. cases			miR-320d		miR-4479		miR-6763-5p	
			Median with interquartile range	*P*-value	Median with interquartile range	*P*-value	Median with interquartile range	*P*-value
Age (year)								
	≥59	82	-1.39 (-2.92 to 1.00)		-2.19 (-3.21 to -1.27)		-3.48 (-5.03 to -1.76)	
	<59	79	-0.97 (-3.13 to 1.34)	0.5522	-2.22 (-3.33 to -0.93)	0.9283	-4.02 (-5.80 to -1.68)	0.3231
BMI								
	≥24	72	-1.30 (-2.93 to 1.11)		-2.23 (-3.30 to -0.75)		-3.64 (-5.22 to -1.76)	
	<24	88	-0.94 (-3.13 to 1.20)	0.7732	-2.07 (-3.22 to -1.15)	0.3527	-3.72 (-5.32 to -1.75)	0.7152
	unknown	1						
Menopause								
	yes	119	-1.01 (-2.91 to 1.25)		-2.07 (-3.21 to -0.97)		-3.71 (-5.17 to -1.95)	
	no	40	-1.01 (-3.36 to 1.07)	0.5392	-2.23 (-3.51 to -1.37)	0.4578	-3.59 (-6.27 to -1.59)	0.6622
	unknown	2						
Tumor position								
	Unilateral	39	-1.31 (-3.69 to 0.98)		-2.32 (-3.17 to -1.67)		-3.38 (-5.32 to -1.68)	
	Bilateral	111	-0.97 (-2.85 to 1.25)	0.3948	-2.07 (-3.36 to -0.97)	0.4604	-3.87 (-5.32 to -1.76)	0.7399
	unknown	11						
Lymph node metastasis								
	yes	83	-0.48 (-2.45 to 1.44)		-1.77 (-3.02 to -0.96)		-3.47 (-5.04 to -1.47)	
	no	65	-2.43 (-3.68 to 0.19)	**0.0008**	-2.59 (-3.63 to -1.75)	**0.0132**	-4.33 (-6.05 to -2.68)	**0.0127**
	unknown	13						
Distant metastasis								
	yes	37	-0.82 (-2.47 to 1.69)		-1.88 (-3.35 to -1.19)		-3.47 (-5.11 to -1.42)	
	no	102	-1.38 (-3.27 to 0.89)	0.0887	-2.23 (-3.21 to -1.10)	0.5507	-3.80 (-5.77 to -1.76)	0.8397
	unknown	22						
FIGO stage								
	I+II	18	-2.86 (-6.57 to -1.22)		-3.03 (-4.39 to -2.15)		-4.49 (-6.72 to -2.83)	
	III	63	0.00 (-2.60 to 1.11)		-1.92 (-2.90 to -0.81)		-3.41 (-5.32 to -1.75)	
	IV	66	-0.87 (-2.90 to 1.60)	**0.0179**	-2.06 (-3.36 to -1.35)	**0.0301**	-3.62 (-5.18 to -1.55)	0.1812
	unknown	14						

Bold values: p<0.05.

### Plasma exosomes isolation

The plasma exosomes were isolated and collected using ultracentrifugation as previously described (21). In short, plasma was centrifuged at 10000×g at 4°C for 30 minutes to remove large vesicles, followed by ultracentrifugation (Beckman Coulter, Brea, CA, USA) at 100,000×g at 4°C for 2 hours to precipitate exosomes. After washing with PBS, exosomes were collected using another ultracentrifugation at 100,000×g at 4°C for 2 hours, and then the exosomes were verified by transmission electron microscopy, qNano and western blot.

### Transmission electron microscopy

For transmission electron microscopy, 15μl exosomes samples were placed on a copper grid, 50 μl 1% glutaraldehyde was fixed on the exosomes for 5 minutes, and then the grid was cleaned with ddH2O for 2 minutes. Next, the grid was placed at 50 μl uranyl oxalate droplets (pH 7, 5 min) and 50ul methylcellulose UA droplets (10 min). A filter paper was used to draw the residual liquids of the grid. Finally, the grid was dried in air for 5 to 10 min. A Tecnai G2 spirit transmission electron microscope (Thermo Fisher, Massachusetts, USA) was used to observe exosomes on the grid.

### Tunable resistive pulse sensing

The separated exosomes were diluted with PBS. The diameter of the exosomes was measured by TRPS and on a qNano platform (Izon Science Ltd, Christchurch, New Zealand). The results were analyzed by Izon control suite v.3.3.2.2000 (Izon Science Ltd).

### Western blotting

Cells and exosomes were lysed by Radioimmunoprecipitation (RIPA) lysis buffer (Beyotime, Jiangsu China) on ice for 30 minutes and then centrifuged at 12000◊g for 15 minutes to obtain protein extracts. All protein samples were quantified by BCA Kit according to the manufacturer’s instructions. Exosomal or cellular protein extracts were separated by SDS-PAGE and then transferred to the PVDF membrane (Billerica millipore, Massachusetts, USA). The membrane was blocked with 5% skimmed milk for 4 hours, and then incubated overnight at 4°C with primary antibodies including anti-GM130, anti-HSP70, and anti-CD9 (CST, Danvers, United State) and secondary antibodies bound to HRP for 1 hour at room temperature. Ultimately, the protein bands were visualized on photographic films using ECL luminescent reagent (bio rad, USA).

### MiRNAs sequencing and data analysis

The exosomal total RNA from 5 EOC patients and 5 healthy donors were extracted and used to construct the library after quality inspection and quantification, including detection of RNA samples integrity by agarose gel electrophoresis and determination of RNA concentration by NanoDrop. Following cluster generation, the library was sequenced for 50 cycles using Illumina Nextseq 500 platform (Illumina, USA). After sequencing, raw sequencing data were subjected to the following preliminary analyzes and processed, including quality control, read mapping to the reference genome, quantitative analysis of miRNAs expression, miRNAs expression difference analysis, target gene prediction, GO and Kyoto Encyclopedia of Genes and Genomes (KEGG) enrichment analysis. R package (3.4.1) was used for differential expression analysis of two conditions/groups, FC (Fold Change) >1.5 and *P*<0.05 were set as the criteria for screening miRNAs. The target genes of miRNAs were predicted using miRDB (http://www.mirdb.org/cgi-bin/search.cgi) and TargetScan (https://www.targetscan.org/vert_80/). GO analysis was performed using top GO package in R environment for statistical computing and graphics. Ingenity Pathway Analysis was used to perform KEGG enrichment analysis.

### RNA isolation and real-time PCR

Exosomal total RNA was extracted by 500 µl TRIzol reagent (Thermo Fisher Scientific, Carlsbad, CA, USA), and then reverse transcribed into cDNA using the Mix-X miRNA First-Strand Synthesis Kit (Accurate Biotechnology, Hunan, China) according to the manufacturer’s instructions. The LightCycle 480 system (Roche, Basel, Switzerland) was used to detect the expression level of exosomal miRNAs, the reaction system includes 2μl of cDNA template, 0.8μl of upstream and 0.8μl of downstream primers, 6.4μl of RNase-free water, and 10μl of SYBR-Green (Accurate Biotechnology, Hunan, China). U6 was used as an internal control (22). The relative gene expression was calculated using ΔCT (CT^miRNAs^-CT^U6)^ as previously described (21). The primer sequences involved are listed in **Table 2**.

**Table 2:** Primers sequence involved.

Gene	Sequence (5′-3′)
U6-Forward primer	GGAACGATACAGAGAAGATTAGC
U6-Reverse primer	TGGAACGCTTCACGAATTTGCG
miR-320d	GAAAAGCTGGGTTGAGAGGAAA
miR-7977	CCCGTGCTCGGAGCAGAAAA
miR-6763-5p	GGGAGTGGCTGGGGAGAAAA

### Statistical analysis

Statistical analysis was performed using GraphPad Prism 8.0 (GraphPad Software, San Diego, CA, USA) and SPSS 25.0 (IBM, Ehningen, Germany) software. The Kolmogorov–Smirnov test was carried out to evaluate the normality of the data distribution. The normally distributed numeric variables were analyzed by parametric test, whereas non-normally distributed variables were evaluated by Mann–Whitney test. One-way ANOVA or Kruskal Wallis one-way ANOVA was used to analyze comparisons among more than two groups. The numerical data were presented in the median and interquartile range. The diagnostic efficiency was evaluated using the receiver operating characteristic curve (ROC). *P*-value < 0.05 was considered statistically significant difference, and all tests were set as double-tailed.

## Results

### Identification of isolated plasma exosomes

**Figure 1:**
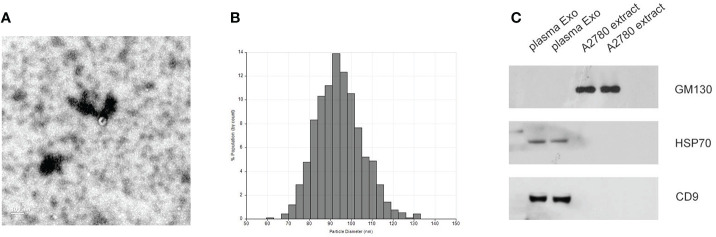
Identification of isolated exosomes. **(A)** TEM image showed the data of exosomes from EOC plasma samples with 50–150 nm diameter. **(B)** Distribution of exosomes with 50–150 nm diameter; the samples were obtained from EOC plasma samples based on the qNano system. **(C)** HSP70, CD9, and GM130 as common exosome markers, were analyzed by western blot.

Plasma exosomes from healthy donors and EOC patients were separated using ultracentrifugation and verified by transmission electron microscopy, qNano, and western blot. As shown in **Figures 1A, B**, the typical exosome-like round morphology with 50–150 nm diameter was observed by TEM, which was consistent with the result of qNano. HSP70 and CD9, as common exosome markers, were significantly enriched in exosomes, while not detected in the cell. Moreover, GM130 (the negative control) was only expressed in the cell but not in exosomes (**Figure 1C**). These results illustrated that the exosomes were successfully isolated using ultracentrifugation.

### Exosomal miRNAs sequencing of the EOC patients

**Figure 2:**
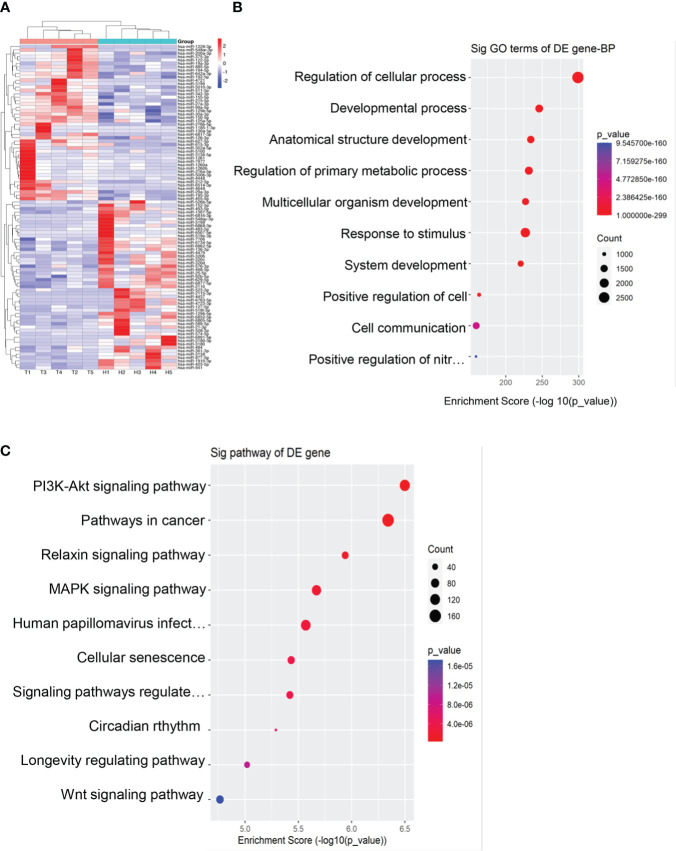
Exosomal miRNAs profile of the EOC patients. **(A)** A heat map was generated after supervised hierarchical cluster analysis. The differential exosomal miRNAs expression is shown in red (upregulation) or blue (downregulation) (*P* < 0.05). **(B)**. Candidate target gene GO enrichment analysis. **(C)** Candidate target gene KEGG enrichment analysis.

We performed miRNA sequencing for plasma exosomes from 5 EOC patients and 5 healthy donors. According to the set standards of *P*<0.05 and >1.5-fold difference between the two groups, 95 miRNAs (50 downregulated and 45 upregulated) were screened (**Figure 2A**). Furthermore, we performed GO and KEGG enrichment analysis for target genes of differentially expressed miRNAs. As shown in **Figures 2B, C**, the target genes of these miRNAs were mainly enriched in the regulation of cellular process, cancer, and PI3K- Akt signaling pathway.

### Exosomal miR-320d, miR-4479, and miR-6763-5p were significantly down-regulated in EOC patients

**Figure 3:**
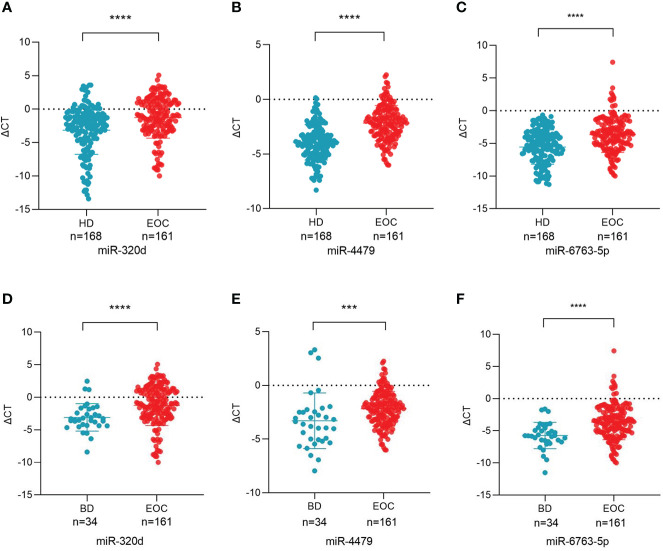
Plasma exosomal miR-320d, miR-4479, and miR-6763-5p are potential biomarkers of EOC. **(A-C)** Scatter plots comparing the exosomal miR-320d, miR-4479, and miR-6763-5p levels in the plasma of healthy donors (n = 168) and EOC patients (n = 161) (****P < 0.0001). **(D-F)** Scatter plots comparing the exosomal miR-320d, miR-4479, and miR-6763-5p levels in the plasma of EOC patients (n = 161) and benign diseases patients (n=34) (***P < 0.001, ****P < 0.0001). HD, Healthy donor; EOC, epithelial ovarian cancer; BD, benign diseases.

Based on primer specificity, 17 miRNAs (9 downregulated and 8 upregulated, **Table 3**) were selected and verified by the small-sample cohort (**Figure S1**). Finally, miR-320d, miR-4479, and miR-6763-5p were identified and further verified in a large-sample cohort. As shown in **Figures 3A-C**, the expression of exosomal miR-320d, miR-4479, and miR-6763-5p were significantly downregulated in EOC patients as compared to healthy controls (*P* < 0.0001 for both), which was consistent with the sequencing results. Moreover, compared with benign disease patients, the levels of three exosomal miRNAs were also decreased significantly in EOC patients (*P* < 0.0001, *P* < 0.001, *P* < 0.0001) (**Figures 3D-F**). This indicated that exosomal miR-320d, miR-4479, and miR-6763-5p have the potential to become novel biomarkers of EOC.

**Table 3:** Up-regulated and down-regulated miRNAs of EOC patients.

miRNA	Fold change	P-value	Description
**hsa-miR-1261**	7.850961264	0.00711725	Up
**hsa-miR-4448**	4.889006818	0.027465476	Up
**hsa-miR-375-3p**	4.458678257	6.06615E-07	Up
**hsa-miR-122-5p**	3.17238141	0.004445662	Up
**hsa-miR-150-5p**	2.642993283	0.001967905	Up
**hsa-miR-1260b**	2.256746925	0.040829104	Up
**hsa-miR-125a-5p**	1.943079621	0.01844784	Up
**hsa-miR-125b-5p**	1.685793721	0.048287292	Up
**hsa-miR-6763-5p**	0.019332181	0.014412069	Down
**hsa-miR-4479**	0.027763541	0.039566672	Down
**hsa-miR-6734-5p**	0.258200206	0.02893196	Down
**hsa-miR-21-3p**	0.318825256	0.036516682	Down
**hsa-miR-320c**	0.42153639	0.000565457	Down
**hsa-miR-320d**	0.475925855	0.0044408	Down
**hsa-miR-320b**	0.505358273	0.005449457	Down
**hsa-miR-2110**	0.533465868	0.015894098	Down
**hsa-miR-7706**	0.612789641	0.044188002	Down

### Characterization of plasma exosomal miR-320d, miR-4479, and miR-6763-5p

**Figure 4:**
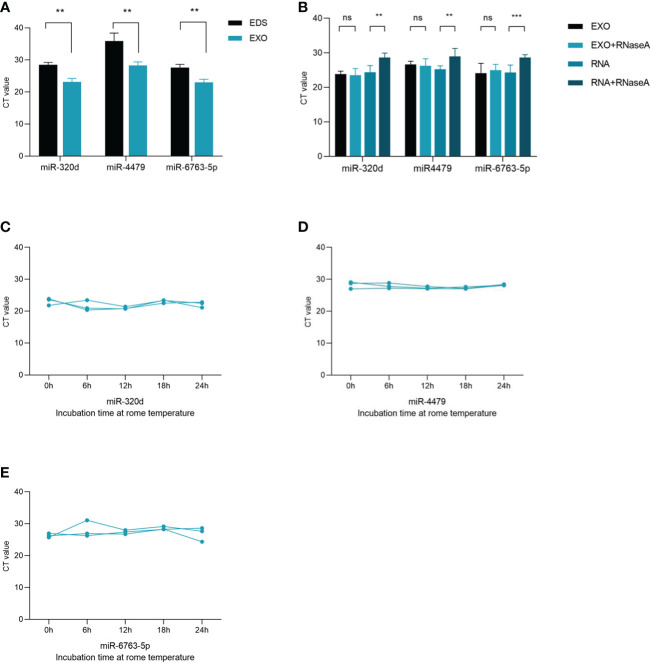
Characterization of identified three plasma exosomal miRNAs. **(A)** Expression levels of miR-320d, miR-4479, and miR-6763-5p from plasma exosomes (EXO) and exosome-depleted supernatant (EDS). **(B)** Expression levels of both miRNAs in exosomes treated with RNase A or in isolated RNA. **(C–E)** The expressions of three plasma exosomal miRNAs when incubated at room temperature (**P < 0.01, ***P < 0.001, ns, not significant).

To confirm whether three miRNAs were specifically distributed into exosomes, we detected the levels of three miRNAs in exosome-depleted supernatant (EDS) and exosomes. The results showed that the expression of three miRNAs in exosomes was significantly higher than that in EDS (**Figure 4A**). Besides, the levels of three miRNAs in exosomes were not significantly changed upon RNase A treatment (**Figure 4B**), which indicated that miRNAs expression was stable in exosomes. In short, these results proved that miR-320d, miR-4479, and miR-6763-5p were included in the exosomes, which prevents miRNAs from being degraded by enzymes. In room temperature incubation test (**Figures 4C-E**), the exosomes were incubated at different time points, such as 0, 6, 12, 18, 24 h, and the levels of three miRNAs did not change significantly.

### Exosomal miR-320d, miR-4479, and miR-6763-5p as biomarkers for EOC

**Figure 5:**
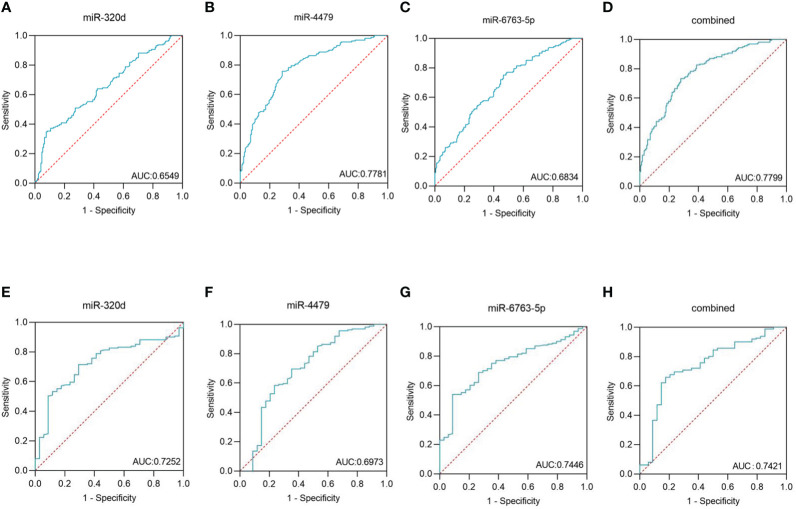
Diagnostic role of plasma exosomal miRNAs expression levels in EOC patients. The AUCs of miR-320d **(A)**, miR-4479 **(B)**, miR-6763-5p **(C)**, and both **(D)** in EOC patients relative to healthy donors. The AUCs of miR-320d **(E)**, miR-4479 **(F)**, miR-6763-5p **(G)** and both **(H)** in EOC patients relative to benign diseases patients. AUC, Areas under the curve.

We used ROC curves to evaluate the diagnostic efficacy of three exosomal miRNAs for EOC. As shown in **Figures 5A-C**, and **Table 4**, the AUC of exosomal miR-320d was 0.6549 (95% CI: 0.596–0.714) with 35.4% sensitivity and 91.7% specificity, the cut-off was 0.535, the AUC of exosomal miR-4479 was 0.7781 (95% CI: 0.728–0.828) with 75.8% sensitivity and 71.4% specificity, the cut-off was -3.225, and the AUC of exosomal miR-6763-5p was 0.6834 (95% CI: 0.627–0.740) with 75.2% sensitivity and 53.6% specificity, the cut-off was -5.255. The combined AUC was 0.7799 with 73.3% sensitivity and 72.0% specificity (**Figure 5D**). Subsequently, ROC curves were employed to evaluate the performance of exosomal miR-320d, miR-4479, and miR-6763-5p as biomarkers for the diagnosis of benign disease patients. As shown in **Figures 5E-G**, and **Table 5**, the AUCs of three exosomal miRNAs were 0.7252 (95% CI: 0.640–0.810) with 71.4% sensitivity and 70.6% specificity, the cut-off was -2.620, 0.6973 (95% CI: 0.588–0.807) with 58.4% sensitivity and 76.5% specificity, the cut-off was -2.455, and 0.7446 (95% CI: 0664–0.826) with 54.0% sensitivity and 91.2% specificity, the cut-off was -3.893, respectively. The combined AUC was 0.7421 with 65.8% sensitivity and 82.4% specificity (**Figure 5H**). The above results indicated that exosomal miR-320d, miR-4479, and miR-6763-5p can act as potential diagnostic biomarkers for EOC.

**Table 4:** Cut-off values of exosomal miR-320d, miR-4479, and miR-6763-5p between healthy donors and EOC patients.

	Optimal Youden index	Sensitivity	Specificity	cut-off values
miR-320d	0.271	35.4%	91.7%	0.535
miR-4479	0.472	75.8%	71.4%	-3.225
miR-6763-5p	0.287	75.2%	53.6%	-5.255

**Table 5:** Cut-off values of exosomal miR-320d, miR-4479, and miR-6763-5p between benign diseases patients and EOC patients.

	Optimal Youden index	Sensitivity	Specificity	cut-off values
miR-320d	0.420	71.4%	70.6%	-2.620
miR-4479	0.349	58.4%	76.5%	-2.455
miR-6763-5p	0.452	54.0%	91.2%	-3.893

### Three exosomal miRNAs are associated with lymph node metastasis and tumor stage

**Figure 6:**
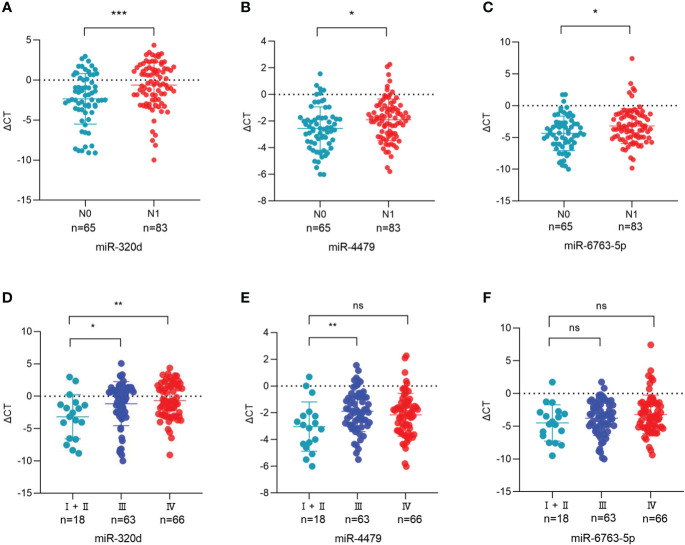
Association between the plasma exosomal miRNAs and the clinical data of EOC patients. **(A-C)** The statistical association between the levels of exosomal miR-320d, miR-4479, and miR-6763-5p and lymph node metastasis. **(D-F)** The plasma exosomal miR-320d, miR-4479, and miR-6763-5p expression in the FIGO stage. (*P <0.05, **P < 0.01, ***P < 0.001, ns: not significant). HD, Healthy donor; EOC, Epithelial ovarian cancer.

The relationship between the expression of exosomal miR-320d, miR-4479, and miR-6763-5p and the clinical data of EOC patients was analyzed (**Table 1**). The results showed that three exosomal miRNAs were not associated with age, BMI, menopause, tumor position, and distant metastasis, but associated with lymph node metastasis. As shown in **Figures 6A-C**, the levels of exosomal miR-320d, miR-4479, and miR-6763-5p were decreased in EOC patients with lymph-node positive as compared to lymph-node negative (*P*=0.0008, *P*=0.0132, *P*=0.0127), then we evaluated the diagnostic efficacy of exosomal miR-320d for lymph node metastasis of EOC, the results showed that the AUC was 0.6602 (95% CI: 0.573–0.748) with 55.4% sensitivity and 70.8% specificity (**Figure S2A**), the AUC of the combination exosomal miR-320d and CA125 was 0.7127 (95% CI: 0.630–0.796) with 60.0% sensitivity and 78.1% specificity (**Figure S2B**), when exosomal miR-320d, CA125, and HE4 were combined, the AUC increased to 0.7367 (95% CI: 0.655–0.818) with 53.8% sensitivity and 85.2% specificity (**Figure S2C**), this indicated that the combination of the three can accurately discriminate EOC patients with positive and negative lymph nodes. Furthermore, we analyzed the relationship between the levels of three exosomal miRNAs and tumor stage, the results showed that exosomal miR-320d and miR-4479 were related to tumor stage, while miR-6763-5p was not related to tumor stage (**Figures 6D-F**).

## Discussion

Although the treatment of ovarian cancer has made great progress, it is difficult to diagnose ovarian cancer, especially early diagnosis, which can be mainly attributed to the lack of effective biomarkers. At present, because of the advantages of noninvasive or minimally invasive sampling and easy operation, liquid biopsy has become a promising method of tumor diagnosis (23). In our study, we screened out exosomal miR-320d, miR-4479, and miR-6763-5p using miRNA sequencing, and verified that three exosomal miRNAs were significantly decreased in EOC patients as compared to healthy controls and benign disease patients, and then evaluated the diagnostic efficiency by ROC curves. The results suggested that exosomal miR-320d, miR-4479, and miR-6763-5p can act as promising biomarkers for EOC.

Recently, many studies had reported that miR-320d can play an important role in a variety of cancers. For example, miR-320d can serve as a promising biomarker of colorectal cancer and inhibit the proliferation and metastasis of EGFR- positive CRC through TUSC3 (24, 25). Serum exosomal miR-320d could be a novel noninvasive biomarker for HCC and the low level of exosomal miR-320d was associated with poor prognosis in HCC patients (26). Furthermore, miR-320d can inhibit KIF3C expression by targeting METTL3 to inhibit the growth and invasion of prostate cancer (27). MiR-320d was also downregulated in breast cancer tissues and can be used as an independent predictor of prognosis in breast cancer, miR-320d and HNF1A-AS1 competitively bind SOX4 to inhibit the progression of breast cancer (28).

In this study, we screened and verified the expression differences of exosomal miR-320d, miR-4479, and miR-6763-5p between the EOC patients and healthy controls. Several pieces of evidence validated that three exosomal miRNAs have the potential as novel biomarkers of EOC. Firstly, exosomal miR-320d, miR-4479, and miR-6763-5p were significantly downregulated in EOC patients as compared to healthy controls and benign disease patients. Secondly, the ROC curves were calculated to assess the diagnostic performance of three exosomal miRNAs for EOC. As a result, the AUC of exosomal miR-320d was 0.6549 with 35.4% sensitivity and 91.7% specificity, the AUC of exosomal miR-4479 was 0.7781 with 75.8% sensitivity and 71.4% specificity, and the AUC of exosomal miR-6763-5p was 0.6834 with 75.2% sensitivity and 53.6% specificity as compared to the healthy controls. Furthermore, the AUCs of three exosomal miRNAs were 0.7252 with 71.4% sensitivity and 70.6% specificity, 0.6973 with 58.4% sensitivity and 76.5% specificity, and 0.7446 with 54.0% sensitivity and 91.2% specificity as compared to the benign disease patients, respectively.

Subsequently, we analyzed the association between the expression levels of exosomal miR-320d, miR-4479, and miR-6763-5p and the clinical characteristics of EOC patients and found that three exosomal miRNAs were related to lymph node metastasis and tumor stage. The levels of exosomal miR-320d, miR-4479, and miR-6763-5p were decreased in EOC patients with lymph node-positive as compared to lymph node-negative, indicating that three exosomal miRNAs may have the potential to predict lymph node metastasis. Taken together, the current data indicated exosomal miR-320d, miR-4479, and miR-6763-5p can act as promising biomarkers for EOC.

Several limitations should be carefully considered in the present study. Firstly, our results included 161 EOC patients and 168 healthy donors, the total sample sizes were small and might lack statistically vigorous power, more samples should be used to further validate the results in this study. Due to the lack of follow-up time, patient survival data could not be obtained to analyze the prognostic value of the three exosomal miRNAs for EOC. Besides, we also did not further study the functions and molecular mechanisms of the three exosomal miRNAs *in vivo* and *in vitro*.

All in all, our results revealed that the levels of exosomal miR-320d, miR-4479, and miR-6763-5p were significantly downregulated in EOC as compared to healthy controls and benign disease patients, processing favorable diagnostic efficiency for EOC. Meanwhile, the levels of three exosomal miRNAs were related to lymph node metastasis and tumor stage of EOC. Therefore, exosomal miR-320d, miR-4479, and miR-6763-5p may be valuable to serve as noninvasive and effective biomarkers for EOC.

## Data Availability

The raw data supporting the conclusions of this article will be made available by the authors, without undue reservation.

## Supplementary material

Supplementary Figure 1 The expression levels of exosomal miRNAs in EOC patients and healthy donors. **(A)** miR-375-3p **(B)** miR-122-5p **(C)** miR-150-5p **(D)** miR-125a-5p **(E)** miR-125b-5p **(F)** miR-320c **(G)** miR-320b **(H)** miR-2110.

Supplementary Figure 2 Diagnostic role of plasma exosomal miR-320d for EOC patients with lymph node metastasis. **(A)** The AUCs of exosomal miR-320d was 0.6602 in lymph node metastatic relative to non-metastatic EOC patients. **(B)** The AUC for the combination of exosomal miR-320d and CA125 was 0.7127 in lymph node metastatic relative to non-metastatic EOC patients. **(C)** The AUC for the combination of exosomal miR-320d, CA125, and HE4 was 0.7367 in lymph node metastatic relative to non-metastatic EOC patients.

## References

[1] Matulonis UA Sood AK Fallowfield L Howitt BE Sehouli J Karlan BY. Ovarian cancer. Nat Rev Dis Primers (2016) 2:16061. doi: 10.1038/nrdp.2016.61 27558151 PMC7290868

[2] Lheureux S Gourley C Vergote I Oza AM. Epithelial ovarian cancer. Lancet (London England) (2019) 393(10177):1240–53. doi: 10.1016/S0140-6736(18)32552-2 30910306

[3] Sung H Ferlay J Siegel RL Laversanne M Soerjomataram I Jemal A. Global cancer statistics 2020: GLOBOCAN estimates of incidence and mortality worldwide for 36 cancers in 185 countries. CA Cancer J Clin (2021) 71(3):209–49. doi: 10.3322/caac.21660 33538338

[4] Chen M Zhao H. Next-generation sequencing in liquid biopsy: Cancer screening and early detection. Hum Genomics (2019) 13(1):34. doi: 10.1186/s40246-019-0220-8 31370908 PMC6669976

[5] Yanez-Mo M Siljander PR Andreu Z Zavec AB Borras FE Buzas EI. Biological properties of extracellular vesicles and their physiological functions. J Extracell Vesicles (2015) 4:27066. doi: 10.3402/jev.v4.27066 25979354 PMC4433489

[6] Liu J Ren L Li S Li W Zheng X Yang Y. The biology, function, and applications of exosomes in cancer. Acta Pharm Sin B (2021) 11(9):2783–97. doi: 10.1016/j.apsb.2021.01.001 PMC8463268 34589397

[7] Kalluri R LeBleu VS. The biology, function, and biomedical applications of exosomes. Science (2020) 367(6478):eaau6977. doi: 10.1126/science.aau6977 32029601 PMC7717626

[8] Bastos N Ruivo CF da Silva S Melo SA. Exosomes in cancer: Use them or target them? Semin Cell Dev Biol (2018) 78:13–21. doi: 10.1016/j.semcdb.2017.08.009 28803894

[9] Brucher BL Jamall IS. Cell-cell communication in the tumor microenvironment, carcinogenesis, and anticancer treatment. Cell Physiol Biochem (2014) 34(2):213–43. doi: 10.1159/000362978 25034869

[10] Valadi H Ekstrom K Bossios A Sjostrand M Lee JJ Lotvall JO. Exosome-mediated transfer of mRNAs and microRNAs is a novel mechanism of genetic exchange between cells. Nat Cell Biol (2007) 9(6):654–9. doi: 10.1038/ncb1596 17486113

[11] Zhou B Xu K Zheng X Chen T Wang J Song Y. Application of exosomes as liquid biopsy in clinical diagnosis. Signal Transduct Target Ther (2020) 5(1):144. doi: 10.1038/s41392-020-00258-9 32747657 PMC7400738

[12] Catalanotto C Cogoni C Zardo G. MicroRNA in control of gene expression: An overview of nuclear functions. Int J Mol Sci (2016) 17(10):1712. doi: 10.3390/ijms17101712 27754357 PMC5085744

[13] Liu X Chen X Yu X Tao Y Bode AM Dong Z. Regulation of microRNAs by epigenetics and their interplay involved in cancer. J Exp Clin Cancer Res (2013) 32:96. doi: 10.1186/1756-9966-32-96 24261995 PMC3874662

[14] Gregory PA Bert AG Paterson EL Barry SC Tsykin A Farshid G. The miR-200 family and miR-205 regulate epithelial to mesenchymal transition by targeting ZEB1 and SIP1. Nat Cell Biol (2008) 10(5):593–601. doi: 10.1038/ncb1722 18376396

[15] Hua S Quan Y Zhan M Liao H Li Y Lu L. miR-125b-5p inhibits cell proliferation, migration, and invasion in hepatocellular carcinoma *via* targeting TXNRD1. Cancer Cell Int (2019) 19:203. doi: 10.1186/s12935-019-0919-6 31384178 PMC6668076

[16] Chang Y Yan W He X Zhang L Li C Huang H. miR-375 inhibits autophagy and reduces viability of hepatocellular carcinoma cells under hypoxic conditions. Gastroenterology (2012) 143(1):177–87 e8. doi: 10.1053/j.gastro.2012.04.009 22504094

[17] Turchinovich A Weiz L Langheinz A Burwinkel B. Characterization of extracellular circulating microRNA. Nucleic Acids Res (2011) 39(16):7223–33. doi: 10.1093/nar/gkr254 PMC3167594 21609964

[18] Thind A Wilson C. Exosomal miRNAs as cancer biomarkers and therapeutic targets. J Extracell Vesicles (2016) 5:31292. doi: 10.3402/jev.v5.31292 27440105 PMC4954869

[19] Sun J Sun Z Gareev I Yan T Chen X Ahmad A. Exosomal miR-2276-5p in plasma is a potential diagnostic and prognostic biomarker in glioma. Front Cell Dev Biol (2021) 9:671202. doi: 10.3389/fcell.2021.671202 34141710 PMC8204016

[20] Zhang Z Tang Y Song X Xie L Zhao S Song X. Tumor-derived exosomal miRNAs as diagnostic biomarkers in non-small cell lung cancer. Front Oncol (2020) 10:560025. doi: 10.3389/fonc.2020.560025 33178588 PMC7592397

[21] Wang L Song X Yu M Niu L Zhao Y Tang Y. Serum exosomal miR-377-3p and miR-381-3p as diagnostic biomarkers in colorectal cancer. Future Oncol (2022) 18(7):793–805. doi: 10.2217/fon-2021-1130 34854318

[22] Sudhakar Reddy P Dhaware MG Srinivas Reddy D Pradeep Reddy B Divya K Sharma KK. Comprehensive evaluation of candidate reference genes for real-time quantitative PCR (RT-qPCR) data normalization in nutri-cereal finger millet [Eleusine coracana (L.)]. *PloS* One (2018) 13(10):e0205668. doi: 10.1371/journal.pone.0205668. eCollection 2018 30321245 PMC6188778

[23] Zheng X Li X Wang X. Extracellular vesicle-based liquid biopsy holds great promise for the management of ovarian cancer. Biochim Biophys Acta Rev Cancer (2020) 1874(1):188395. doi: 10.1016/j.bbcan.2020.188395 32698041

[24] Yufeng Z Ming Q Dandan W. MiR-320d inhibits progression of EGFR-positive colorectal cancer by targeting TUSC3. Front Genet (2021) 12:738559. doi: 10.3389/fgene.2021.738559 34733314 PMC8558375

[25] Liu X Xu X Pan B He B Chen X Zeng K. Circulating miR-1290 and miR-320d as novel diagnostic biomarkers of human colorectal cancer. J Cancer (2019) 10(1):43–50. doi: 10.7150/jca.26723 30662524 PMC6329864

[26] Li W Ding X Wang S Xu L Yin T Han S. Downregulation of serum exosomal miR-320d predicts poor prognosis in hepatocellular carcinoma. J Clin Lab Anal (2020) 34(6):e23239. doi: 10.1002/jcla.23239 32125733 PMC7307335

[27] Ma H Zhang F Zhong Q Hou J. METTL3-mediated m6A modification of KIF3C-mRNA promotes prostate cancer progression and is negatively regulated by miR-320d. Aging (Albany NY) (2021) 13(18):22332–44. doi: 10.18632/aging.203541 PMC8507285 34537760

[28] Shi S Hu X Xu J Liu H Zou L. MiR-320d suppresses the progression of breast cancer *via* lncRNA HNF1A-AS1 regulation and SOX4 inhibition. RSC Adv (2018) 8(34):19196–207. doi: 10.1039/C8RA01200H PMC9080600 35539662

