# Supplementary figures and images for: Plasma exosomal miR-320d, miR-4479, and miR-6763-5p as diagnostic biomarkers in epithelial ovarian cancer
Source: Front Oncol. 2022 Dec 14;12:986343. doi: 10.3389/fonc.2022.986343 (PMC9795228; doi:10.3389/fonc.2022.986343)

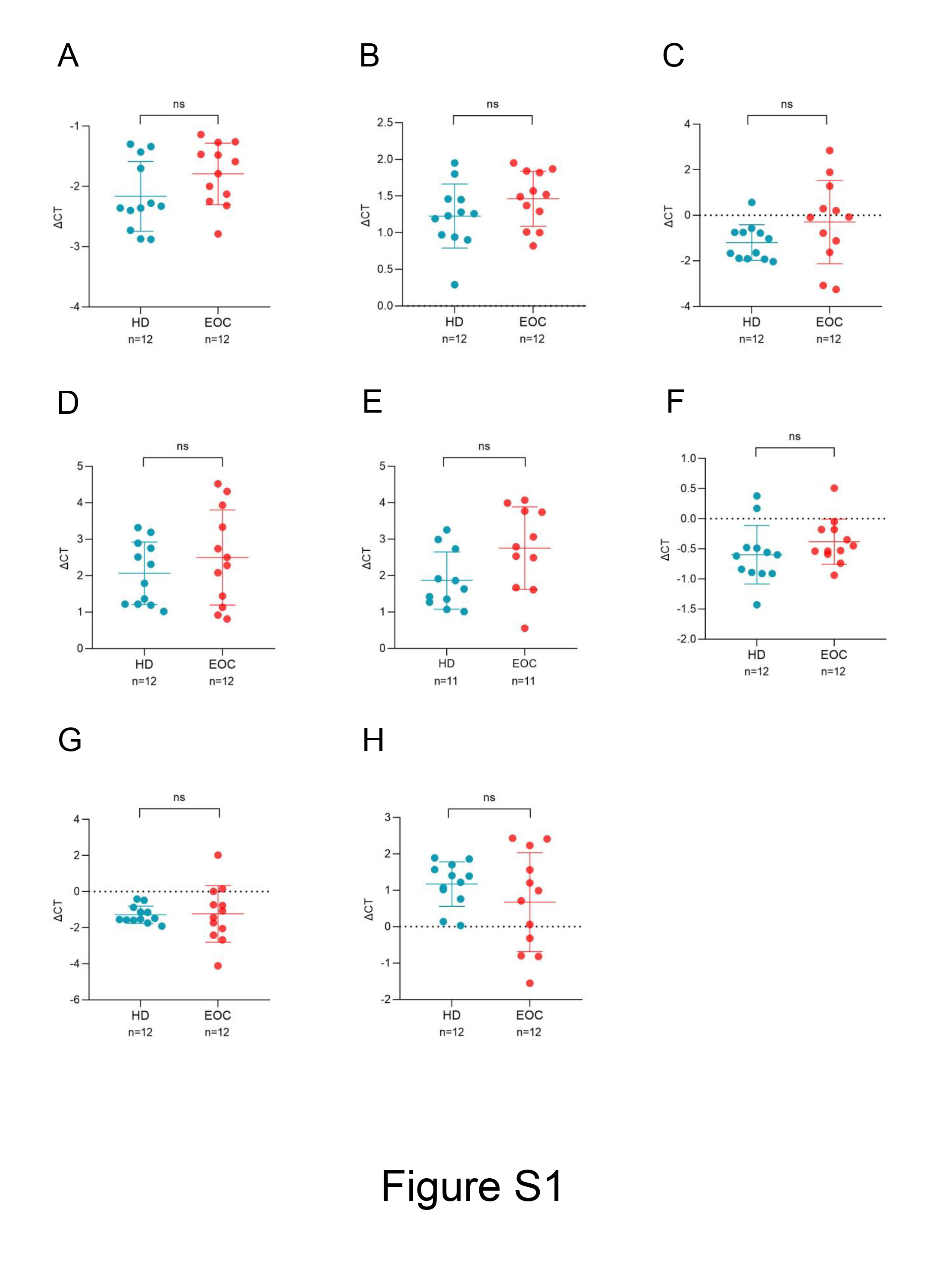

Supplement: Supplementary Figure 1 — The expression levels of exosomal miRNAs in EOC patients and healthy donors. (A) miR-375-3p (B) miR-122-5p (C) miR-150-5p (D) miR-125a-5p (E) miR-125b-5p (F) miR-320c (G) miR-320b (H) miR-2110. [file Image_1.jpeg]

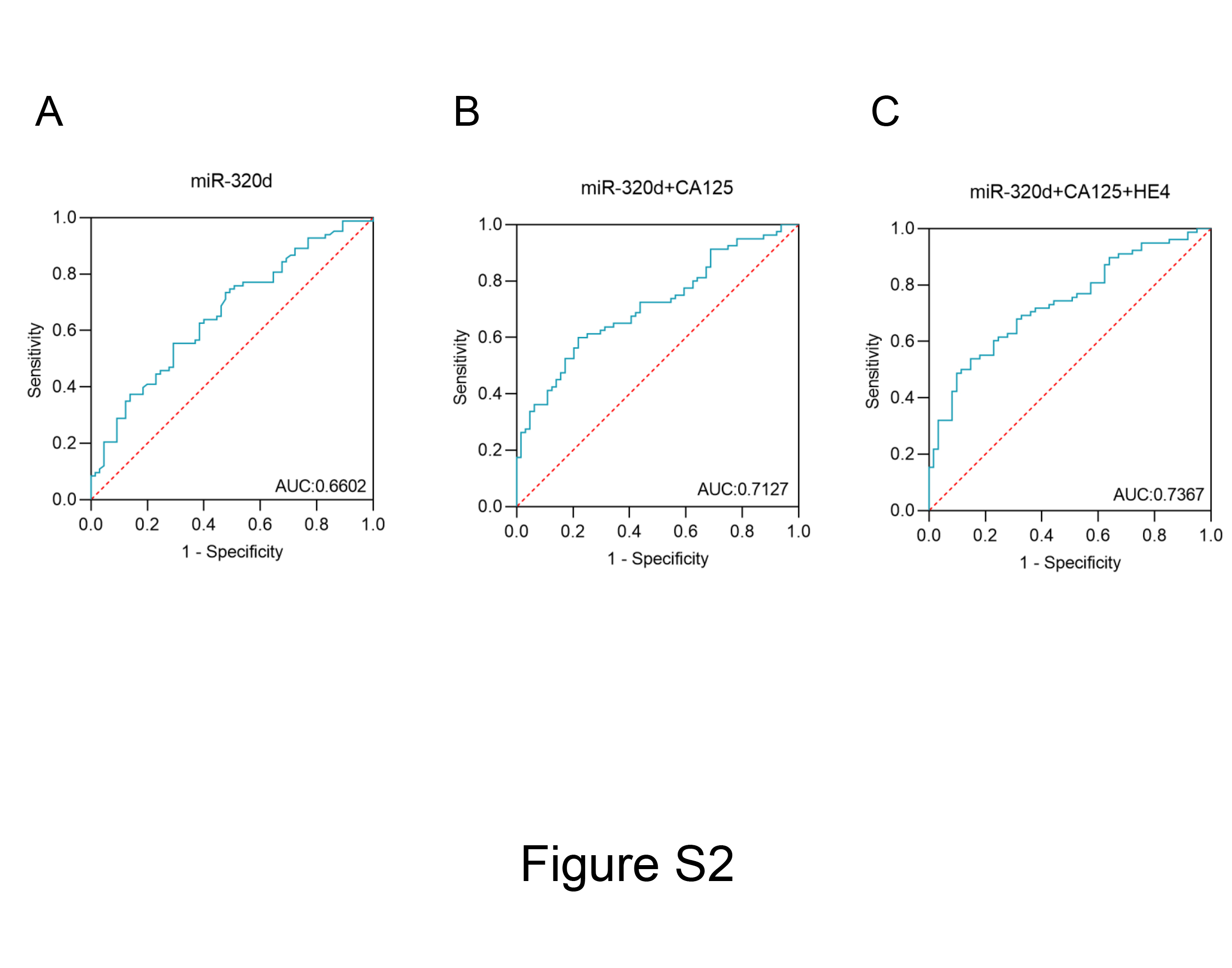

Supplement: Supplementary Figure 2 — Diagnostic role of plasma exosomal miR-320d for EOC patients with lymph node metastasis. (A) The AUCs of exosomal miR-320d was 0.6602 in lymph node metastatic relative to non-metastatic EOC patients. (B) The AUC for the combination of exosomal miR-320d and CA125 was 0.7127 in lymph node metastatic relative to non-metastatic EOC patients. (C) The AUC for the combination of exosomal miR-320d, CA125, and HE4 was 0.7367 in lymph node metastatic relative to non-metastatic EOC patients. [file Image_2.jpeg]
